# *ASPM*-lexical tone association in speakers of a tone language: Direct evidence for the genetic-biasing hypothesis of language evolution

**DOI:** 10.1126/sciadv.aba5090

**Published:** 2020-05-27

**Authors:** Patrick C. M. Wong, Xin Kang, Kay H. Y. Wong, Hon-Cheong So, Kwong Wai Choy, Xiujuan Geng

**Affiliations:** 1 Department of Linguistics and Modern Languages, The Chinese University of Hong Kong, Shatin, Hong Kong.; 2 Brain and Mind Institute, The Chinese University of Hong Kong, Shatin, Hong Kong.; 3 Department of Otorhinolaryngology, Head and Neck Surgery, The Chinese University of Hong Kong, Shatin, Hong Kong.; 4 School of Biomedical Sciences, The Chinese University of Hong Kong, Shatin, Hong Kong.; 5 Department of Obstetrics and Gynaecology, The Chinese University of Hong Kong, Shatin, Hong Kong.

## Abstract

How language has evolved into more than 7000 varieties today remains a question that puzzles linguists, anthropologists, and evolutionary scientists. The genetic-biasing hypothesis of language evolution postulates that genes and language features coevolve, such that a population that is genetically predisposed to perceiving a particular linguistic feature would tend to adopt that feature in their language. Statistical studies that correlated a large number of genetic variants and linguistic features not only generated this hypothesis but also specifically pinpointed a linkage between *ASPM* and lexical tone. However, there is currently no direct evidence for this association and, therefore, the hypothesis. In an experimental study, we provide evidence to link *ASPM* with lexical tone perception in a sample of over 400 speakers of a tone language. In addition to providing the first direct evidence for the genetic-biasing hypothesis, our results have implications for further studies of linguistic anthropology and language disorders.

## Teaser

A link between *ASPM* gene and tone feature provides direct evidence for the genetic-biasing hypothesis of language evolution.

## INTRODUCTION

According to a recent estimate, there are currently 7111 known living languages in the world (*1*). These languages are diverse in structural features (*2*) such as word order (e.g., English, but not Korean, follows a canonical subject-verb-object order) and sound (e.g., Cantonese, but not English, uses pitch to contrast word meaning). Insights into the factors that modulate these typological differences have implications for the origin of language, human evolution, and population genetics. One controversial, hypothesized driving factor of typological differences and language evolution is genetic diversity at the population level. Specifically, it has been postulated that a linguistic feature appears or is retained in a language because the population that speaks that language is genetically predisposed to an enhanced ability to process that feature (*3*, *4*). Thus far, evidence for this genetic-biasing hypothesis comes from statistical analysis of the correlation between genetic variation and linguistic typological variation at the population level (*3*) and, to some extent, computational modeling of gene and language coevolution (*5*). As fundamental as these studies are in formulating the genetic-basing hypothesis, direct evidence of linkage between genes and linguistic features has yet to emerge. The present study aims to collect this direct evidence with human participants. If individual linguistic features cannot be shown to be influenced by genetic variations, the genetic-biasing hypothesis cannot be established.

In generating the genetic-biasing hypothesis, Dediu and Ladd (*3*) analyzed a large database of 983 alleles and 26 linguistic features in 49 populations. Notably, they found a significant correlation between two alleles and lexical tone, even after controlling for geographical and historical factors. Lexical tones are pitch patterns that are used by speakers to contrast word meaning. Languages that use lexical tones, called tone languages, comprise at least half of the world’s languages (*6*) and are more concentrated in Central Africa and East Asia. Dediu and Ladd (*3*) found that populations that have a lower frequency of the derived allele of two genes that are associated with microcephaly, *ASPM* (rs41310927) and *MCPH1* (rs930557), are more likely to speak a tone language.

Studies of the molecular and cellular functions of *ASPM* and *MCPH1*, mostly focusing on their orthologs and homologs in *Drosophila* and mice, have revealed their roles in mitosis (*7*–*8*). Both genes are expressed in the cerebral cortical ventricular zone and proliferative zones (*9*) and play crucial roles in normal neurogenesis (*10*–*12*). Loss-of-function mutations of both genes cause primary microcephaly (*9*, *13*, *14*). Curiously, although the derived alleles of *ASPM* and *MCPH1* are specific to humans and have been implicated in the adaptive evolution of the human brain (*15*–*16*), earlier experimental attempts failed to find connections of these allelic variations in overall brain size either in adults (*17*–*18*) or in children (*19*). A more recent study found the effect of *ASPM* mutations on neuroanatomical measures to be nonuniform across regions of the brain, with the auditory cortex [transverse temporal gyrus (also known as Heschl’s gyrus) and the lateral surface of the temporal lobe] being more affected than the structures of the medial temporal lobe (*20*). These neuroanatomical findings in respect of the auditory cortex converge with the lexical tone findings by Dediu and Ladd (*3*), as the pitch center in primates is located in the Heschl’s gyrus (*21*). A smaller volume of the Heschl’s gryus is associated with poorer lexical tone learning in European Americans who had no prior knowledge of a tone language (*22*). Furthermore, in a small-scale study of 32 participants, polymorphisms of *ASPM* (rs41310927) were found to be associated with pitch pattern perception in European Americans who did not speak a tone language (see Discussion for details of this study) (*23*).

Because of feasibility and ethical concerns of testing human participants, evolutionary studies often rely on inductive reasoning based on the deployment of advanced analytics that can handle large datasets. Inductive reasoning provides an important first step in formulating a hypothesis of gene-behavior associations of evolutionary significance. To more definitively test these gene-behavior associations, it has been argued that deductive approaches of hypothesis-driven experimentations are needed (*17*). Although fundamental in proposing the genetic-biasing hypothesis of language evolution with evidence from a gene-tone association from inductive reasoning (*3*), direct experimental evidence of the hypothesized tone perception function of *ASPM* is still lacking.

In the present study, we use a hypothesis-driven approach in our study of microcephaly gene-tone association. If our hypothesis is correct, it would provide direct evidence for the genetic-biasing hypothesis of language evolution (*3*, *4*). On the basis of findings of previous studies, we hypothesize that genes that are associated with microcephaly, especially the exonic single-nucleotide polymorphism (SNP) rs41310927 of *ASPM*, are expressed in the pitch center of the auditory cortex, and their polymorphisms lead to differential pitch perceptual abilities across individuals. Because of their pitch perception functions, populations with a specific pattern of polymorphisms of microcephaly-related genes would be more likely to exploit pitch in their language, as was indicated by the Dediu and Ladd study (*3*).

We tested over 400 adult native speakers of Cantonese, a tone language that has an inventory of six lexical tones, one of the largest tonal inventories of all tone languages (*24*). The participants were asked to perform lexical tone, music (rhythm and pitch), and general cognitive tasks (Table 1). Information about their musical background was also obtained. In addition to microcephaly-related genes that have been implicated in previous studies [especially *ASPM* (rs41310927)], we also examined a number of genes that have been hypothesized to be related to general cognitive and language processing. This design, involving multiple tasks and multiple genes, allows us to ascertain the specificity of the hypothesized gene-tone association.

**Table 1:** Demographic information and phenotype scores of participants. M, male; F, female; NA, not applicable.

**Variables**	**Range**	**Mean**	**Total *N* of** **cases**
Age (years)	18–27	20.84	426
Gender	95 (M); 331 (F)	NA	426
IQ	85–130	108.13	419
Music year	0–20	6.18	423
Lexical tone	0.51–1.00	0.87	412
Musical pitch	0.46–1.00	0.77	426
Rhythm	0.17–1.00	0.84	426
Running working memory	0.05–1.00	0.62	408

## RESULTS

We examined the association between lexical tone perception and SNPs of a group of microcephaly-related genes whose hypothesized functions concern the brain (*20*, *25*, *26*), pitch (*23*), and lexical tone (Table 2) (*3*). As one control procedure, we examined the association of these SNPs with three other auditory-based behaviors (musical pitch perception, rhythm perception, and auditory working memory). Of particular interest is *ASPM* (rs41310927) because of the two previous studies that implicate its tone- and pitch-related functions (*3*, *23*). As another control procedure, we examined the associations between the four auditory phenotypes with a group of genes that are associated with (*28*–*30*) or hypothesized to be associated with (*31*) broad language functions or disorders (henceforth “control SNPs”; see table S1). Genotype-phenotype associations were examined via the convergence of the general linear model (GLM) and machine learning prediction analysis.

**Table 2:** SNPs of microcephaly-related genes that are hypothesized to be associated with lexical tone perception. Information for the alleles is obtained from the latest dbSNP database published by the National Center for Biotechnology Information (United States) for East Asians or based on our current sample.

**Gene**	**SNP**	**Population**	**Phenotype**	**Allele** **frequency** **in dbSNP** **database**	**Allele** **frequency** **in our** **sample**	***N* of cases for each genotype** **in our sample**			**Total *N*** **of** **cases**	**References**
*MCPH1*	rs1057090	Han Chinese	Cranial volume	T = 0.672; C = 0.328	T = 0.698; C = 0.302	TT = 210	TC = 172	CC = 42	424	(*26*)
*MCPH1*	rs11779303	European (Norwegian)	Cortical area	G = 0.966; C = 0.034	G = 0.960; C = 0.040	GG = 390	GC = 34	CC = 0	424	(*25*)
*MCPH1*	rs930557	European (Norwegian)	Pitch perception	C = 0.764; G = 0.236	C = 0.754; G = 0.246	CC = 240	CG = 159	GG = 25	424	(*3*, *23*)
*MCPH1*	rs2816517	European (Norwegian)	Brain volume	C = 0.824; A = 0.176	C = 0.800; A = 0.200	CC = 277	CA = 124	AA = 23	424	(*25*)
*ASPM*	rs41310927	European (Norwegian)	Pitch perception	T = 0.836; C = 0.164	T = 0.838; C = 0.162	TT = 299	TC = 113	CC = 12	424	(*3*, *23*)
*CDK5RAP2*	rs1888893	European (Norwegian)	Cortical area	A = 0.855; G = 0.145	A = 0.863; G = 0.137	AA = 310	AG = 96	GG = 9	415	(*25*)
*CDK5RAP2*	rs4836819	European (Norwegian)	Cortical area	G = 0.818; A = 0.182	G = 0.808; A = 0.192	GG = 275	GA = 135	AA = 14	424	(*25*)
*CDK5RAP2*	rs7859743	European (Norwegian)	Cortical area	G = 0.815; A = 0.185	G = 0.807; A = 0.193	GG = 273	GA = 138	AA = 13	424	(*25*)
*CDK5RAP2*	rs914592	European (Norwegian)	Cortical area	A = 0.897; G = 0.103	A = 0.871; G = 0.129	AA = 323	AG = 93	GG = 8	424	(*25*)

### General linear model

We constructed separate multiple linear regression models for each of the four different auditory behaviors (dependent variables), using the microcephaly-related SNPs, age, gender, intelligence quotient (IQ), and years of musical training as independent variables. For lexical tone perception, a significant model fit was found (adjusted *R*^2^ = 0.168, *P* < 0.001), with IQ (corrected *P* = 0.007), years of musical training (corrected *P* < 0.001), and the minor allele load of rs41310927 (corrected *P* = 0.039) showing significant associations after correction for multiple comparisons (Table 3). No significant model fit was found for musical pitch (table S2). A significant model fit was found for rhythm (adjusted *R*^2^ = 0.032, *P* = 0.018), with IQ (uncorrected *P* = 0.008) and years of musical training (uncorrected *P* = 0.017) showing significant associations (neither survived after correction for multiple comparisons) (see table S3). The model for auditory working memory (table S4) was not significant. After correction for multiple comparisons, no significant associations were found for any SNPs with any auditory behaviors, except for rs41310927 and lexical tone perception. There were no significant associations found in multiple linear regression models using the control SNPs as independent variables and any of the four auditory behaviors as dependent variables (tables S5 to S8).

**Table 3:** Summary of the multiple linear regression model with lexical tone as the dependent variable. Variables listed in the first column are independent variables. *R*^2^ = 0.195 (adjusted *R*^2^ = 0.168); *P* < 0.001 (this model).

	**β**	***P***	**Corrected** ***P***	**Partial η^2^**
**Age**	−0.005	0.200	0.520	0.004
**Gender**	−0.011	0.371	0.689	0.002
**IQ**	0.002	<0.001*‡	0.007	0.031
**Music year**	0.007	<0.001*‡	<0.001	0.117
**rs1057090**	0.019	0.068	0.221	0.009
**rs11779303**	−0.004	0.819	0.887	0.000
**rs930557**	−0.005	0.636	0.827	0.001
**rs2816517**	−0.004	0.708	0.837	0.000
**rs41310927**	−0.029	0.009*‡	0.039	0.018
**rs1888893**	−0.033	0.509	0.827	0.001
**rs4836819**	−0.026	0.613	0.885	0.001
**rs7859743**	0.047	0.354	0.767	0.002
**rs914592**	0.012	0.826	0.826	0.000


*P < 0.05 (uncorrected).

‡Significant associations after FDR corrections for multiple comparisons.

**Fig. 1:**
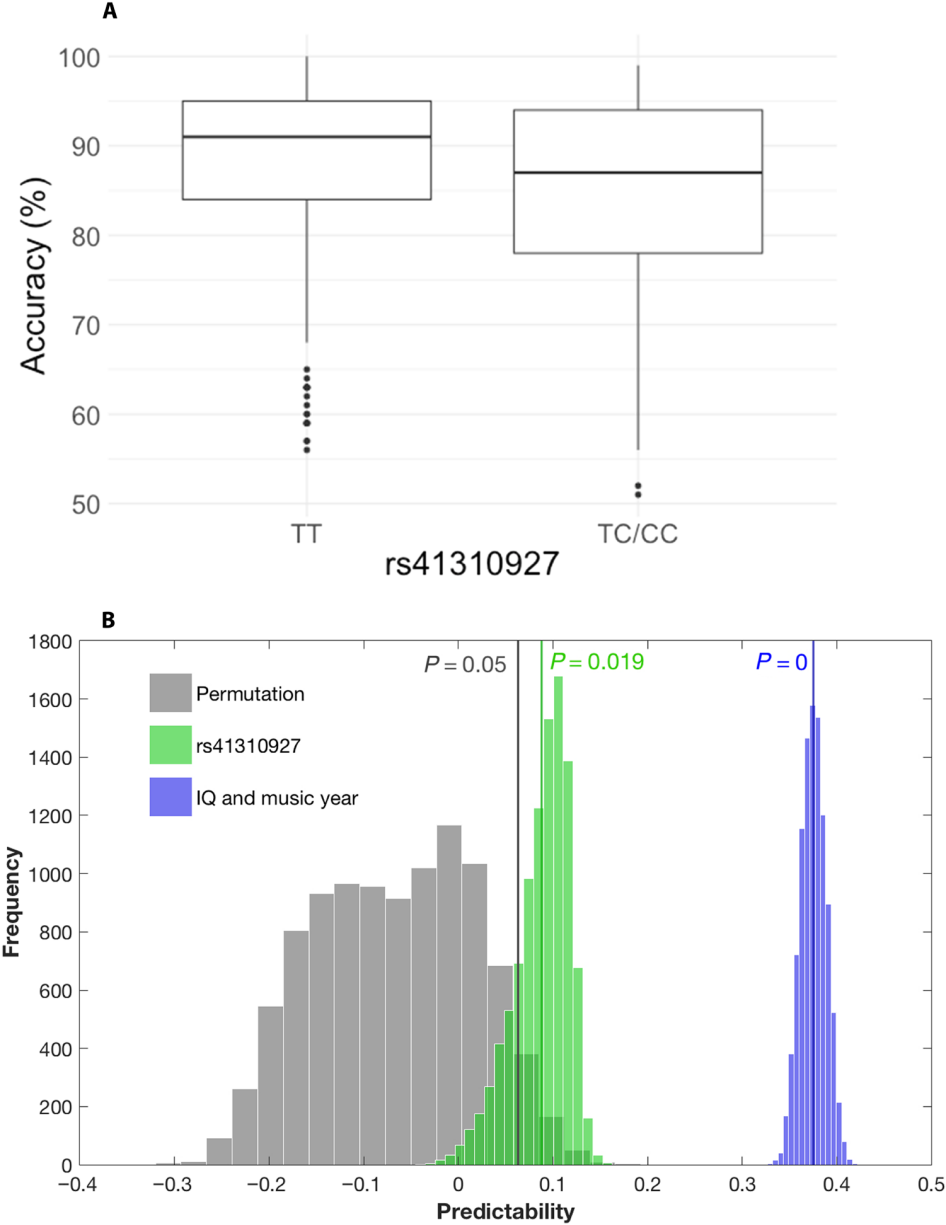
Association between lexical tone perception and SNP rs41310927. (**A**) Mean accuracy of lexical tone perception of TT carriers (mean = 87.68%, SD = 10.17) is significantly higher than TC/CC carriers (mean = 83.99%, SD = 12.01, *t* = 2.97, *P* = 0.003, Cohen’s *d* = 0.33). (**B**) Prediction of tone perception using predictors showing significant associations with tone perception using SVR. The predictability was estimated by the correlation coefficients (cc) between the predicted and the observed tone perception scores. Results show that with only SNP rs41310927, the distribution of prediction values (green) was significantly different (*P* = 0.019) from the null distribution (gray). With IQ and years of musical training, the distribution of prediction value (blue) was much higher and was also significantly different (*P* < 0.001) from the null distribution.

We then constructed a stepwise regression model for lexical tone with the same set of independent variables (all of the hypothesized SNPs, age, gender, IQ, and years of musical training) as the multiple linear regression models discussed above. Years of musical training with IQ contributed to the first two models that explained up to 15.6% of variance in lexical tone perception. The final significant model had rs41310927 explaining another 1.4% of variance (adjusted *R*^2^ = 0.170) (Table 4). Figure 1A shows lexical tone performance by the rs41310927 allele groups.

**Table 4:** Summary of the stepwise regression model with lexical tone as the dependent variable. Age, gender, IQ, and SNPs listed in Table 2 are independent variables.

**Model**		**Unstandardized coefficients**		**Standardized** **coefficients**	***t***	**Sig.**	**Adjusted *R***^**2**^
		B	SE	β			
**1**	(Constant)	0.820	0.008		103.386	<0.001	0.131
	Music year	0.007	0.001	0.364	7.728	<0.001	
**2**	(Constant)	0.633	0.053		12.013	<0.001	0.156
	Music year	0.007	0.001	0.340	7.243	<0.001	
	IQ	0.002	<0.001	0.169	3.601	<0.001	
**3**	(Constant)	0.613	0.053		11.626	<0.001	0.170
	Music year	0.007	0.001	0.328	7.015	<0.001	
	IQ	0.002	<0.001	0.169	3.639	<0.001	
	rs41310927	0.030	0.011	0.125	2.708	0.007	

### Machine learning prediction analysis

The aforementioned GLM analysis results all suggested a significant association between rs41310927 and lexical tone perception, along with IQ and years of musical training. To obtain converging evidence from multiple types of analytics, we used machine learning [support vector regression (SVR)]. Predictability is estimated by the Pearson correlation coefficients (cc) between predicted and observed lexical tone performance. The higher the cc values are, the more accurate the predictive performance of the SVR model is. With IQ and years of musical training as predictors, the predicted cc (mean = 0.375) was significantly different from the null distribution (*P* < 0.001) (Fig. 1B). With only SNP rs41310927 as the predictor, the distribution of predicted cc (mean = 0.088) was also significantly different (*P* = 0.019) from the null distribution, although as expected, the predicted cc with one SNP was much lower than when the other two variables were used as predictors.

To summarize, in both GLM and machine learning analyses, we found musical training along with IQ to be the most significant contributors of lexical tone performance, as predicted by previous studies (*32*, *33*). Although the contribution was much smaller, rs41310927 was significantly associated with lexical tone perception, after the effects of IQ and musical training were accounted for, with the minor allele C contributing negatively to lexical tone perception. A convergence of analytics confirmed these findings. No other significant associations were found between any other SNPs and lexical tone, or any SNPs with any auditory behaviors examined. The association between rs41310927 seems to be specific and is unrelated to general auditory functions. The odds ratios (ORs) for an rs41310927 C allele carrier performing below the mean or 1 SD below the mean on the lexical tone perception task are 2.13 [95% confidence interval (CI), 1.38 to 3.29] and 1.90 (95% CI, 1.11 to 3.26), respectively.

## DISCUSSION

In a hypothesis-driven manner, our study provides the first evidence linking a genetic variant with a linguistic feature, as far as we are aware, providing the most direct support for the genetic-biasing hypothesis of language evolution to date. The association between the genetic variant that we studied, *ASPM* (rs41310927), and the lexical tone perception has already been implicated in two previous studies. The first one was a population-level correlational study (*3*) that did not examine the function of the gene but was nevertheless foundational in generating the current hypothesis. The second was a small-scale study that linked nonlexical pitch perception with this variant in non–tone-speaking European Americans (see below for discussion of potential discrepancies between this study and the present study) (*23*). The present study is the third study to examine *ASPM* (rs41310927) and the first experimental study to examine the genetic impact of *ASPM* (rs41310927) on a large group of native tone language speakers perceiving lexical tone. The significant association remained even after musical experience and IQ were controlled for. Although we are not claiming an exclusive association, our results suggest that the association between this genetic variant and lexical tone is quite specific. That is, this association seems to be related to perceiving pitch patterns within a syllable-level time frame and holding that pitch information across two to three syllables. The variant is not associated with general musical abilities that require attending sequences of notes, pitch, or rhythm in a longer, phrasal context. It is also not associated with general auditory working memory. Lexical tone perception is also not associated with other genes that have been implicated in general language functions (e.g., *CNTNAP2*).

A number of previous studies have examined the association between genes and language-related phenotypes. For example, one study found an association between *CNTNAP2* and non–word repetition and developmental language disorder more broadly (*28*). A previous study identified *FOXP2* and a severe form of verbal apraxia (*34*). These studies provided strong evidence of the genetic basis of general language and cognitive skills and disorders, but they did not refer specifically to a linguistic feature. Likewise, Stein *et al*. (*35*) studied the association between variants of *ASPM* (excluding rs41310927, which we studied here) and a number of broad language skills. In contrast to the studies of *CNTNAP2* and *FOXP2*, Stein *et al*. (*35*) did not find any significant associations between *ASPM* and general language behaviors.

It is important to emphasize that support for the genetic-biasing hypothesis must include findings that link linguistic features, rather than general auditory, language, or cognitive functions, with genes. Essentially, this hypothesis explains how different languages have evolved to lead to these diverse sets of linguistic features and presents a genetic explanation for their evolution. Population-level differences in allele frequencies of certain genes may change the probability of associations with specific linguistic features. This change in association may provide part of an explanation for some typological differences of languages. In our case, the frequency of the T and C alleles of rs41310927 has been reported to be around 59 and 41% for Europeans (*36*) and 84 and 16% in our sample of over 400 Han Chinese, respectively. Our study reveals that the T allele enhances lexical tone perception abilities in Han Chinese who speak a tone language as their native language. Note that other genetic variants that we studied also showed differences in allele frequencies across populations, but none were significantly associated with lexical tones. For example, the frequency of the major allele in East Asian and European populations are, respectively, 72 and 50% for *COMT* (rs4680), 94 and 46% for *DRD2* (rs6277), and 73 and 52% for *ATP2C2* (rs11860694). This further indicates a specific relationship between rs41310927 and lexical tone.

Note that by far the strongest modulating effect of lexical tone perception was musical experience. This is in line with previous research suggesting a connection between music and lexical tone. We found that musicians who were native English-speaking adults with no experience of a tone language were able to learn a tone language better (*32*), and their auditory system encoded lexical tones more accurately (*37*) than their counterparts with little or no musical training. Cantonese speakers were also better at musical pitch (but not rhythm) perception than French and English speakers, after the musical and educational background was controlled for, presumably due to their experience of speaking a tone language (*27*). Caldwell-Harris *et al*. (*33*) found that heritage speakers of a tone language were able to notice pitch differences in a new tone language in the “exposure phase” of an implicit learning experiment better than Asian learners without a tone language background. Their results suggest that auditory and language experience played a larger role in lexical tone learning than genetics. Nevertheless, even after musical experience was accounted for, our results suggest that the influence of genetics cannot be altogether discounted. As expected, the effect size was small and within the limits of the variance attributable to a single SNP as far as human cognitive abilities are concerned. Okbay *et al*. (*38*) found that the 74 SNPs that they examined together explained only 4% of the variance in educational attainment (an average of 0.05% variance explained). After the contribution of musical experience and IQ, rs41310927 explained about 1.4% of the variance in lexical tone perception, which is substantially more than the average SNP documented by Okbay *et al*. (*38*). We do not expect the *ASPM* SNP that we examined to be the only SNP associated with lexical tone nor did we expect it to not contribute to any other functions. The significance of the current findings is that we have provided the strongest evidence to date connecting a gene and a linguistic feature and that this association is unlikely to be of a general purpose nature. Our results are also in line with previous gene-brain research that found *APSM* to be expressed in the auditory cortex (*20*, *23*).

Although the present study and our previous, smaller-scale study (*23*) both found a significant association between lexical (pitch) perception and *ASPM*, the association was in opposite directions. Most notably, the direction of the current findings is consistent with the direction hypothesized by Dediu and Ladd (*3*) for native tone speakers. There are at least two explanations for this discrepancy. First, as explained in Wong *et al*. (*23*), the same allele could exert a different phenotypic effect due to gene-gene and culture-gene interactions (*39*–*41*). The participants in Wong *et al*. (*23*) were non–tone-speaking European Americans, whereas the participants in the present study were tone-speaking Han Chinese. Second, as argued by Caldwell-Harris *et al*. (*33*), the association between *ASPM* and lexical tone concerns a phenotype requiring the listeners to integrate pitch with the rest of the syllable. On the contrary, the AX task used by Wong *et al.* (*23*) required the non–tone-speaking participants to perform the opposite, namely, segregating rather than integrating pitch with the syllable. This explanation is further supported by previous studies showing tone language speakers’ inability to ignore tonal differences when a task required them to only attend to segmental information, a phenomenon known as segmental-suprasegmental integration (*42*, *43*). To further boost tonal integration in the syllable, the present study incorporated an ABX task, which required the participants to retain tonal information within a syllable for a longer period of time in memory. This change in task, as well as the tendency of tone language speakers (but not non–tone language speakers) to integrate pitch with the syllable, may explain the discrepancy between the present study and our previous study (*23*). Further research is required to disentangle whether *ASPM*-tone association is related to more basic pitch perception or whether it is specific to higher-level phonological pitch processing. As native and nonnative listeners approach tasks of different levels of suprasegmental processing differently (e.g., AX versus ABX) even in nontone suprasegmental domains such as stress (*44*–*46*), future research should carefully consider the interaction between task demand and language background of the participants to understand the specific phenotype being investigated and its association with genes. In the present study, we use the term “perception” to describe our task, because the participants were not explicitly required to conduct phonological or lexical tone processing.

Besides providing direct evidence for the genetic-biasing hypothesis of language evolution, the present study may also have clinical and educational implications. In the case of tone language speakers, poor tone perception is associated with a number of communication disorders, including developmental language disorder (*47*) and autism spectrum disorder (ASD) (*48*). As indicated by the ORs, carriers of the C allele of *ASPM* (rs41310927) have a higher risk of lower tone perception performance. The OR of about 2 that we obtained is comparable to or higher than those individual SNPs that have the highest ORs for Alzheimer’s disease (*49*) and ASD (*50*). Thus, future research might investigate whether *ASPM* is a candidate gene for communication disorders and whether it is a genetic marker specific to individuals who speak a tone language (but not individuals who speak a nontone language). In addition to being a linguistic feature, lexical tone perception may be an endophenotype of the more complex language disorders. Studies of endophenotypes have proven to be fruitful in the study of the genetic basis of schizophrenia (*51*–*52*) and ASD (*53*) and are also believed to be promising for language as well (*54*).

**Fig. 2:**
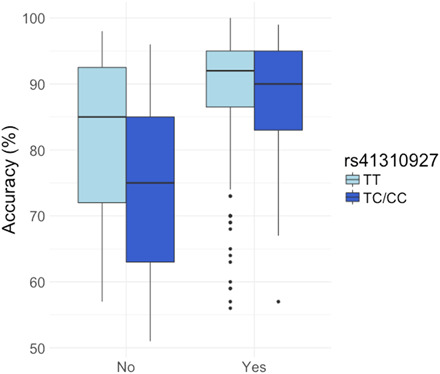
Lexical tone perception performance depends on an interaction between genotype and musical training. The *x* axis indicates participants without musical training and with at least 1 year of musical training, separated by allele group of rs41310927. A significant music × gene interaction was found [*F*(404) = 7.28, *P* = 0.007].

Our findings may also have implications for personalized learning (*55*). In an exploratory (post hoc) analysis, we investigated the potential differential modulating effects of musical training on lexical tone in carriers of the T and C alleles. As the T allele favored tone perception, it is possible that musical training would be less likely to improve the tone perception of the T allele carriers. As shown in Fig. 2, musical training seems to interact with genotype, so that C allele carriers seem to have benefited more from musical training, as far as the tone-enhancing effect of musical training is concerned. The implication for personalized learning is that carriers of the C allele should consider receiving musical training to potentially prevent a tone perception disorder and to enhance learning. Future research should be specifically designed to further investigate this hypothesis.

In conclusion, we found direct evidence of gene-tone association, providing the critical direct evidence for the genetic-biasing hypothesis of language evolution. We hypothesize that *ASPM* is expressed in the pitch center of the auditory cortex (*20*), which would, in turn, enhance the lexical tone perception of carriers of the favored allele. Populations with a higher frequency of the favored allele would be more likely to have lexical tone in their language. In addition to the two previous studies implicating the role of *ASPM* in pitch and tone perception (*3*, *23*), our results are also consistent with a computational modeling study of language evolution, suggesting that tone is less likely to be affected by historical and geographical factors and more likely to be influenced by speaker-internal factors in its evolution (*4*). Future studies might fruitfully attempt to identify more tone-related genes and also genes for other linguistic features to provide further support for the genetic-biasing hypothesis. Future studies may also consider how genetic and environment factors (*56*) interact in driving typological differences.

## MATERIALS AND METHODS

### Participants

A total of 426 (331 females) individuals were recruited. All participants were reported to be native speakers of Cantonese of Han Chinese decent without hearing, neurological, or psychiatric disorders. Informed consent was obtained from each participant. The age of the participants ranged from 18 to 27 years with an average age of 20.82 years (SD = 1.44). We estimated the nonverbal intelligence (henceforth IQ) of each participant by using the Test of Nonverbal Intelligence (Fourth Edition) (*57*); all participants scored within normal limits. All participants also passed a hearing screening at 25 dB HL (decibels in hearing level) for the frequencies of 250, 1000, 2000, and 4000 Hz in a sound booth. Each participant reported how many years of musical training they had received, if any. Not every measure contained data from all participants. Tables 1 and 2 and table S1 state how many participants were included in each measure. Reasons for missing data included DNA quality, inability to complete all tasks due to fatigue, incomplete data submission by the participants, and coding errors. Our sample size was determined by assuming the use of independent two-sample *t* tests for behavioral differences across two genotypic groups. We assumed the minor allele frequency to be 0.3 (in our current sample, 29% of the participants had at least one minor allele of rs41310927). The effect size was estimated by examining three relevant published studies (*23*, *25*, *26*), ranging from 0.26 to 0.84. The mean effect size of 0.47 was used in our sample size estimation. With nine hypothesized SNPs and four phenotypes, the Bonferroni-corrected alpha was 0.0014 (uncorrected α = 0.05). With 13 control SNPs and four phenotypes, the Bonferroni corrected alpha was 0.00096. Therefore a corrected alpha of 0.001 was used in our final estimation. According to this estimation, 372 participants were required to achieve the detection power of 80%. Thus, our final sample size of 426 participants allowed us to detect potentially significant results even with missing data of up to 15%. The research protocol was approved by the Joint Chinese University of Hong Kong–New Territories East Cluster Clinical Research Ethics Committee.

### Behavioral measures

All participants were tested on two pitch-related and two non–pitch-related behavioral auditory experiments to examine domain generality and specificity of the hypothesized genotype-phenotype associations. The experiments were administered using a Windows laptop with a screen size of 15 inches. Participants were instructed to wear a Sennheiser headphone during the study. They were offered a 5-min break after the completion of each task. The order of the tasks was counterbalanced.

#### Lexical tone perception

In an ABX task, participants were asked to judge in each three-syllable trial whether the tone of the last syllable was the same as the first or the second syllable. The last syllable matched the first/second syllable in 50% of the trials. Lexical tone was the only dimension that varied across the three syllables in a trial. All syllables were pseudowords (words that obeyed Cantonese phonological rules but had no meaning) in Cantonese. The syllables were originally produced by a native female speaker of Cantonese in her mid-20s. These syllables were subsequently resynthesized to normalize duration and amplitude. Each syllable was 400 ms long. In total, there were 204 trials. The task lasted for approximately 15 min. Chance performance level was 50%.

#### Musical pitch perception

In each trial, participants listened to a pair of melodies composed in the Western tonal (6 trials), pentatonic (6 trials), and atonal (12 trials) scales. In 50% of the trials, the two melodies in each pair were completely identical. In the other 50%, the melodies were almost identical, except that one note had a different pitch to its counterpart. The participants were asked to indicate whether the two melodies were identical. Each melody lasted about 11 s. In total, there were 24 trials. The task lasted for approximately 12 min. Chance performance level was 50%.

#### Rhythm perception

This task closely followed the musical pitch task, except for the different trials, where the melodies differed in time (rhythm) rather than in musical pitch. The melodies were composed in the Western tonal (six trials) and pentatonic (six trials) scales. Each melody lasted for approximately 11 s. In total, there were 12 trials. The task lasted for approximately 8 min. Chance performance level was 50%.

#### Running working memory

We adapted an auditory working memory task (*58*). The participants listened to trials of three to seven letters (D, F, J, K, L, N, P, Q, and R) that were presented sequentially with a 200-ms interstimulus interval in a randomized order. The letters were normalized for intensity and duration (600 ms). After all letters were presented, a response screen appeared and reminded the participants how many letters to report. The responses of the task were constrained only to a forward order. Participants were required to recall the letters in the exact sequence as the order of presentation. They typed the letters in the response box on their screens using a keyboard. In total, the participants finished 15 trials including 75 letters in total. The task lasted 15 min. One point was given for each item correctly recalled in the correct position of the trial. For example, if four letters—D, N, F, and R—were to be reported, participants who responded “D, Q, F, and R” would receive 3 points, but those who responded “D, F, and R” would receive only 1 point. Participants’ performance was aggregated as a percentage of correct recalls.

### Genes and SNP genotyping

The genomic DNA was extracted from saliva samples, collected using Oragene kits (DNA Genotek). Extracted DNA samples were quantified by a NanoDrop spectrophotometer and normalized to 5 ng/μl for use in genotyping. Genotyping of the SNPs was conducted using a commercially available Sequenom MassARRAY platform (*59*). The pattern of allele frequencies of our sample is consistent with that reported by the dbSNP database published by the National Center for Biotechnology Information (United States) (https://www.ncbi.nlm.nih.gov/snp/) (see Table 2 and table S1) (*36*). We examined two groups of SNPs. The hypothesized, microcephaly-related SNPs have been hypothesized to be related to brain, pitch, and/or lexical tone functions (Table 2) (*3*, *20*, *23*, *25*, *26*). The control SNPs are hypothesized to be related to general language functions, especially grammatical functions, because of their relationship with the neurotransmitter dopamine and, potentially, the corticostriatal pathway (*30*, *31*) or because they have been shown repeatedly to be related to language impairment, motor speech disorder, and/or specifically the phenotype of non–word repetition (table S1) (*28*, *29*). These control SNPs are not hypothesized to be associated directly with lexical tone or non–language-related auditory behaviors, such as those being examined. The linkage disequilibrium among the SNPs on the same chromosome was examined with PLINK (fig. S3) (*60*).

### Association between SNP genotypes and behavioral measures

We conducted a series of analyses to evaluate the hypothesis that the specific microcephaly-related SNPs (Table 2) are associated with lexical tone perception. As a control procedure, we examined whether the same microcephaly-related SNPs are associated with other auditory phenotypes. As a final control procedure, we investigated whether a group of genes (table S1) hypothesized to be related to language is associated with these phenotypes. Correlation and GLM analyses were conducted, followed by machine learning for the significant SNP to provide converging results.

#### General linear statistical models

SNP-wise multiple linear regression analyses were conducted to test whether the effect of the SNPs was associated with each of the four behavioral measures. Two sets of models were built, one for each of the microcephaly-related genes and control (language-related) genes. The SNPs were coded as binary genotypes: homozygous carriers of the major alleles coded as 0, and carriers with at least one minor allele coded as 1. Note that for the hypothesized SNPs, less than 10% of the participants were homozygous for the minor allele; thus, we determined to combine them with those participants who had at least one minor allele. The genotypes served as the independent variables, and each of the behavioral measures was dependent variables. Age, gender, IQ, and years of musical training were entered simultaneously in each of the multiple linear regression model for each dependent variable. Gender was coded as 0 (male) and 1 (female) in the model. The false discovery rate (FDR) based on Benjamini and Hochberg was used for correcting the multiple comparisons. Stepwise regression analyses were conducted after performing multiple linear regression (“METHOD = stepwise” in SPSS). This enabled us to further examine whether adding some of the predictor(s) can significantly improve the prediction of the behavioral measures.

#### Machine learning prediction analysis

We further conducted prediction analyses using the significant associated regressors as predictors to test whether tone perception can be predicted by these predictors. A nested *k*-fold leave-one-out cross-validation (LOOCV) procedure with an SVR classifier under support vector machine was used for prediction (*61*). In the inner level with *k*-1 folds, optimized parameters were selected. Linear kernel with penalty parameter C = 1 and ε = 0.001 was selected and used for the outer level. The parameters were then applied to the outer level of one fold for testing. A permutation test by randomly ordering the observed data was computed for generating a null distribution. The permutation procedure was repeated 10,000 times with *k*-fold LOOCV to generate the null distributions of the model performance. The 5% percentile was set as the critical value for a one-tailed *t* test of the null hypotheses (prediction accuracy being the same as random predictions).

## Supplementary Material

aba5090_SM.pdf

## SUPPLEMENTARY MATERIALS

Supplementary material for this article is available at http://advances.sciencemag.org/cgi/content/full/6/22/eaba5090/DC1

## Appendix

View/request a protocol for this paper from *Bio-protocol*.
