# Supplementary material for: ASPM-lexical tone association in speakers of a tone language: Direct evidence for the genetic-biasing hypothesis of language evolution
Source: Sci Adv. 2020 May 27;6(22):eaba5090. doi: 10.1126/sciadv.aba5090 (PMC7253162; doi:10.1126/sciadv.aba5090)
Supplement: aba5090_SM.pdf [file aba5090_SM.pdf]

Science Advances
AAAS

advances.sciencemag.org/cgi/content/full/6/22/eaba5090/DC1

Supplementary Materials for

ASPM-lexical tone association in speakers of a tone language: Direct evidence for the genetic-biasing hypothesis of language evolution

Patrick C. M. Wong*, Xin Kang, Kay H. Y. Wong, Hon-Cheong So, Kwong Wai Choy, Xiujuan Geng

*Corresponding author. Email: p.wong@cuhk.edu.hk

Published 27 May 2020, *Sci. Adv.* **6**, eaba5090 (2020)
DOI: 10.1126/sciadv.aba5090

This PDF file includes:

Additional Methods
Additional Results
Tables S1 to S10
Figs. S1 to S3
References

Additional Methods

Lexical tone perception. The lexical tone perception task consists of these Cantonese tonal pairs: T1T2, T1T3, T1T4, T1T5, T2T3, T2T4, T2T5, T3T4, T3T5, T3T6, T4T5, and T5T6.

Correlations between genes and behaviours. Non-parametric correlations were calculated between the loads of minor alleles of each SNP and each of the behavioural scores. The Spearman's correlations were estimated using IBM SPSS Statistics v22. Partial correlations controlling for the effect of age, gender, IQ and years of musical training were also calculated to estimate the associations between genotypes and each of the behavioural measures. The same analyses were conducted on both the hypothesised and controlled SNPs. As these are first-level analysis, allele loads for each participant were coded 0, 1, or 2 depending on the number of minor allele present.

Additional Results

Correlations between demographic variables and performance on the four behavioural tasks. The distributions of the four behavioural measures are shown in Figure S1. Pair-wise non-parametric (Spearman's) correlation tests were conducted between demographic and behavioural measures including age, gender, IQ, years of musical training and four behavioural measures (Figure S2). After correction for multiple comparisons, these measures were significantly correlated with each other: IQ vs. tone (r=.224, p=5 x 10^{-6}, corrected p=1.4 x 10^{-4}), music year vs. tone (r=.348, p=3.93 x 10^{-13}, corrected p=1.1 x 10^{-11}) and rhythm (r=.154, p=1.49 x 10^{-3}, corrected p=0.0417), tone vs. musical pitch (r=0.179, p=2.7 x 10^{-4}, corrected p=7.6 x 10^{-3}), rhythm (r=0.293, p=1.417 x 10^{-9}, corrected p=3.97 x 10^{-8}), and working memory (r=0.18, p=2.95 x 10^{-4}, corrected p=8.3 x 10^{-3}), and musical pitch vs. rhythm (r=0.338, p=7.26 x 10^{-13}, corrected p=2.03 x 10^{-7}).

Correlations between genes and behaviours. Both non-parametric (Spearman's) correlation tests and partial correlation tests (controlling for age, gender, IQ and years of musical training) between the genotypes and behavioural measures reported a significant correlation between the minor allele load of *ASPM* (rs41310927) and tone perception, after FDR correction for multiple comparison corrections (Table S9 and S10). The corrected p value of the Spearmen's correlation was .036, and the corrected partial p value was .045. No other significant correlations were found between any other SNP loads and any behavioural measures. There were no significant correlations (after FDR correction for multiple comparisons) observed between the control SNPs and any behavioural measures using both non-parametric or partial correlation analyses. These first-level results converge with the GLM and machine-learning results presented in the article.

Table S1. Control SNPs related to the dopamine system, language functions and/or disorders. Alleles are listed based on the latest dbSNP database published by the National Center for Biotechnology Information (US) for East Asians or are based on our current sample.

Gene	SNP	Population	Phenotype	Allele frequency in dbSNP database	Allele frequency in our sample	N of cases for each genotype in our sample			Total N of Cases	References
CNTNAP2	rs10246256	European (UK	Expressive and receptive language abilities (E & R); Non-word repetition (NR)	T=.653; C=.347	T=.683; C=.317	TT=178	TC=223	CC=23	424	28
CNTNAP2	rs2538976	Han Chinese	Speech sound disorder	T=.574; C=.426	T=.560; C=.440	TT=137	TC=201	CC=86	424	62
CNTNAP2	rs2538991	European (UK)	E&R; NR	C=.631; A=.369	C=.640; A=.360	CC=166	CA=211	AA=47	424	28
CNTNAP2	rs2710102	Han Chinese	Speech sound disorder	G=.633; A=.367	G=.640; A=.360	GG=166	GA=211	AA=47	423	62
CNTNAP2	rs2710117	European (Australian)	E & R; NR	A=.641; T=.359	A=.652; T=.348	AA=172	AT=209	TT=43	424	63
CNTNAP2	rs759178	European (Australian)	E & R; NR	C=.632; A=.368	C=.640; A=.360	CC=166	CA=211	AA=47	424	63
CNTNAP2	rs851715	European (Australian);	E & R; NR	T=.645; C=.355	T=.652; C=.348	TT=171	TC=211	CC=42	424	63
ATP2C2	rs11860694	Europeans (UK)	Phonological short-term memory; dyslexia;	G=.727; C=.273	G=.738; C=.262	GG=228	GC=170	CC=26	424	29
DRD1	rs4532	Various	Executive Function	T=.846; C=.154	T=.846; C=.154	TT=309	TC=99	CC=16	424	31, 64
DRD2	rs6277	European (American); European (Italian)	Working memory	G=.938; A=.062	G=.926; A=.074	GG=366	GA=53	AA=5	424	65, 66, 67
COMT	rs4680	European (Spanish); Han Chinese	Hippocampal volume; brain dopamine level breakdown	G=.720; A=.280	G=.715; A=.285	GG=211	GA=176	AA=31	418	68, 69, 70
CMIP	rs6564903	European (UK)	Reading related traits; dyslexia short term memory	T=.795; C=.205	T=.804; C=.196	TT=273	TC=134	CC=16	423	28
FOXP2	rs6980093	Han Chinese	Speech production; sensorimotor control	A=.574; G=.426	A=.626; G=.374	AA=163	AG=196	GG=58	417	71

Table S2. Summary of the multiple linear regression model with musical pitch as the dependent variable and the hypothesised SNPs as independent variables. Variables listed in the first column are independent variables. R-squared = .052 (Adjusted R-squared = .020); p of this model = .072. * indicates p < .05 (uncorrected); ‡ represents significant associations after FDR corrections for multiple comparisons.

	Beta	p	Corrected p	Partial Eta Squared
**Age**	.002	.616	1.001	.001
**Gender**	-.005	.692	.900	.000
**IQ**	.000	.735	.796	.000
**Music Year**	.003	.003*‡	.039	.023
**rs1057090**	-.002	.851	.851	.000
**rs11779303**	-.035	.083	.360	.008
**rs93055**	.015	.204	.663	.004
**rs2816517**	-.026	.030	.195	.012
**rs41310927**	-.005	.704	.832	.000
**rs1888893**	.048	.397	1.032	.002
**rs4836819**	.027	.631	.911	.001
**rs7859743**	-.044	.441	.956	.002
**rs914592**	-.033	.577	1.072	.001

Table S3. Summary of the multiple linear regression model with rhythm as the dependent variable and the hypothesised SNPs as independent variables. Variables listed in the first column are independent variables. R-squared = .063 (Adjusted R-squared = .032); p of this model = .018. * indicates p < .05 (uncorrected).

	Beta	p	Corrected p	Partial Eta Squared
**Age**	.001	.792	1.030	.000
**Gender**	.034	.060	.260	.009
**IQ**	.002	.008*	.104	.018
**Music Year**	.003	.017*	.111	.014
**rs1057090**	.000	.984	.984	.000
**rs11779303**	-.037	.162	.301	.005
**rs930557**	.017	.256	.370	.003
**rs281651**	.004	.798	.943	.000
**rs41310927**	.003	.869	.941	.000
**rs1888893**	.113	.125	.325	.006
**rs4836819**	-.135	.071	.231	.008
**rs7859743**	.115	.127	.275	.006
**rs914592**	-.100	.194	.315	.004

Table S4. Summary of the multiple linear regression model with working memory as the dependent variable and the hypothesised SNPs as independent variables. Variables listed in the first column are independent variables. R-squared = .041 (Adjusted R-squared = .007); p of this model = .259.

	Beta	p	Corrected p	Partial Eta Squared
**Age**	.005	.570	0.926	.001
**Gender**	-.026	.341	0.887	.002
**IQ**	.001	.313	1.356	.003
**Music Year**	.002	.309	2.009	.003
**rs1057090**	-.022	.317	1.030	.003
**rs11779303**	.023	.584	.759	.001
**rs930557**	.008	.736	.797	.000
**rs2816517**	-.054	.023	.299	.014
**rs41310927**	-.013	.580	.838	.001
**rs1888893**	-.069	.528	.981	.001
**rs4836819**	-.038	.736	.870	.000
**rs7859743**	-.023	.836	.836	.000
**rs914592**	.090	.433	.938	.002

Table S5. Summary of the multiple linear regression model with lexical tone as the dependent variable and the control SNPs as independent variables. Variables listed in the first column are independent variables. The list of control SNPs was used as independent variables (see Table S1). R-squared = .182 (Adjusted R-squared = .149); p of this model < .001. Due to the identical distribution of allele frequencies for SNPs rs253891, rs2710102 and rs759178, the parameters for rs2710102 and rs759178 were not estimated, similarly for Table S6, S7, and S8.

	Beta	p	Corrected p	Partial Eta Squared
**Age**	-.005	.156	.468	.005
**Gender**	-.013	.322	.690	.003
**IQ**	.002	<.001	.003	.033
**Music Year**	.007	.000	.000	.120
**rs10246256**	-.034	.404	.758	.002
**rs2538976**	.004	.750	.804	.000
**rs2538991**	.053	.155	.581	.005
**rs2710102**	.	.	.	.
**rs2710117**	-.056	.087	.435	.008
**rs759178**	.	.	.	.
**rs851715**	.042	.292	.730	.003
**rs11860694**	.001	.933	.933	.000
**rs4532**	.006	.630	.788	.001
**rs6277**	-.010	.540	.900	.001
**rs4680**	.004	.691	.797	.000
**rs6564903**	-.006	.559	.839	.001
**rs6980093**	-.005	.609	.831	.001

Table S6. Summary of the multiple linear regression model with musical pitch as the dependent variable and the control SNPs as independent variables. Variables listed in the first column are independent variables. The list of control SNPs was used as independent variables (see Table S1). R-squared = .054 (Adjusted R-squared = .017); p of this model = .122.

	Beta	p	Corrected p	Partial Eta Squared
**Age**	.002	.560	.764	.001
**Gender**	-.005	.744	.930	.000
**IQ**	.000	.524	.786	.001
**Music Year**	.003	.007	.105	.019
**rs10246256**	.064	.159	.397	.005
**rs2538976**	.004	.788	.909	.000
**rs2538991**	.095	.024	.180	.013
**rs2710102**	.	.		.
**rs2710117**	-.083	.024	.120	.013
**rs759178**	.	.		.
**rs851715**	-.073	.102	.382	.007
**rs11860694**	-.017	.151	.453	.005
**rs4532**	-.009	.465	.775	.001
**rs6277**	.002	.899	.899	.000
**rs4680**	.008	.463	.868	.001
**rs6564903**	.002	.889	.952	.000
**rs6980093**	.009	.457	.979	.001

Table S7. Summary of the multiple linear regression model with rhythm as the dependent variable and the control SNPs as independent variables. Variables listed in the first column are independent variables. The list of control SNPs was used as independent variables (see Table S1). R-squared = .078 (Adjusted R-squared = .042); p of this model =.07.

	Beta	p	Corrected p	Partial Eta Squared
**Age**	.000	.928	.928	.000
**Gender**	.028	.124	.372	.006
**IQ**	.002	.012	.090	.016
**Music Year**	.003	.041	.153	.011
**rs10246256**	.143	.016	.080	.015
**rs2538976**	-.014	.404	.551	.002
**rs2538991**	.063	.243	.521	.004
**rs2710102**	.	.	.	.
**rs2710117**	-.066	.166	.415	.005
**rs759178**	.	.	.	.
**rs851715**	-.168	.004	.060	.022
**rs11860694**	.011	.455	.569	.001
**rs4532**	-.015	.364	.607	.002
**rs6277**	.021	.350	.656	.002
**rs4680**	-.007	.643	.742	.001
**rs6564903**	.014	.386	.579	.002
**rs6980093**	-.006	.669	.717	.000

Table S8. Summary of the multiple linear regression model with working memory as the dependent variable and the control SNPs as independent variables. Variables listed in the first column are independent variables. The list of control SNPs was used as independent variables (see Table S1). R-squared = .037 (Adjusted R-squared = -.002); p of this model = .512.

	Beta	p	Corrected p	Partial Eta Squared
**Age**	.006	.445	.834	.002
**Gender**	-.038	.175	1.312	.005
**IQ**	.001	.422	.904	.002
**Music Year**	.002	.407	1.018	.002
**rs10246256**	-.085	.336	1.008	.003
**rs2538976**	-.003	.899	.899	.000
**rs2538991**	.032	.707	.964	.000
**rs2710102**	.	.	.	.
**rs2710117**	.040	.588	.882	.001
**rs759178**	.	.	.	.
**rs851715**	.013	.877	.940	.000
**rs11860694**	.048	.035	.525	.012
**rs4532**	-.030	.232	1.160	.004
**rs6277**	-.035	.318	1.193	.003
**rs4680**	.014	.522	.870	.001
**rs6564903**	.004	.854	.985	.000
**rs6980093**	.005	.834	1.043	.000

Table S9 Partial correlations between the minor allele loads of the hypothesised SNPs and behavioural measures, controlling for age, gender, IQ and years of musical training. * indicates $p < 0.05$ (uncorrected); ‡ represents significant correlations after FDR corrections of multiple comparisons. The p values reported here are uncorrected.

**SNP**	**Tone**		**Musical pitch**		**Rhythm**		**Working memory**	
	**Partial correlation**	**p**	**Partial correlation**	**p**	**Partial correlation**	**p**	**Partial correlation**	**p**
rs1057090	.069	.178	-.003	.956	.012	.812	-.074	.147
rs11779303	-.018	.727	-.080	.108	-.079	.115	.049	.340
rs930557	.011	.825	.055	.275	.064	.203	.072	.159
rs2816517	.015	.771	-.105	.035	-.020	.684	-.105	.040*
rs41310927	-.142	.005*‡	-.018	.715	.032	.525	.003	.957
rs1888893	.077	.130	.026	.609	.004	.932	-.079	.121
rs4836819	.094	.065	.009	.858	-.014	.786	-.093	.068
rs7859743	.098	.055	-.003	.959	-.001	.978	-.098	.055
rs914592	.045	.382	.032	.524	-.003	.960	-.065	.203

Table S10. Non-parametric Spearman's correlations between minor allele loads of the hypothesised SNPs and behavioural measures. * indicates p < .05 (uncorrected); ‡ represents significant correlations after FDR corrections of multiple comparisons. The p values reported here are uncorrected.

**SNP**	**Tone**		**Musical pitch**		**Rhythm**		**Working memory**	
	**Spearman's rho**	**p**	**Spearman's rho**	**p**	**Spearman's rho**	**p**	**Spearman's rho**	**p**
rs1057090	.012	.817	-.019	.702	-.017	.733	-.092	.066
rs11779303	-.042	.393	-.099	.045*	-.083	.092	.032	.521
rs930557	-.034	.496	.059	.232	.039	.433	.016	.756
rs2816517	.034	.494	-.110	.024*	.002	.960	-.093	.065
rs41310927	-.141	.004*‡	-.030	.544	.005	.924	-.035	.488
rs1888893	.014	.784	.032	.510	.020	.683	-.045	.374
rs4836819	.020	.693	-.007	.890	-.011	.826	-.057	.258
rs7859743	.020	.683	-.018	.720	.001	.976	-.061	.222
rs914592	.030	.546	.027	.578	.008	.872	-.038	.448

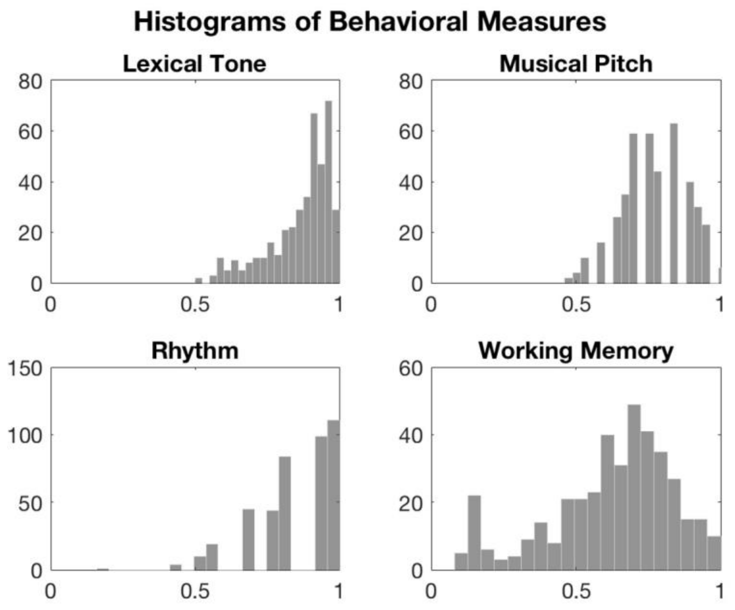

Fig. S1. Histograms of performance of the four behavioural tasks. The tasks include lexical tone perception, musical pitch perception, rhythm perception, and running working memory.

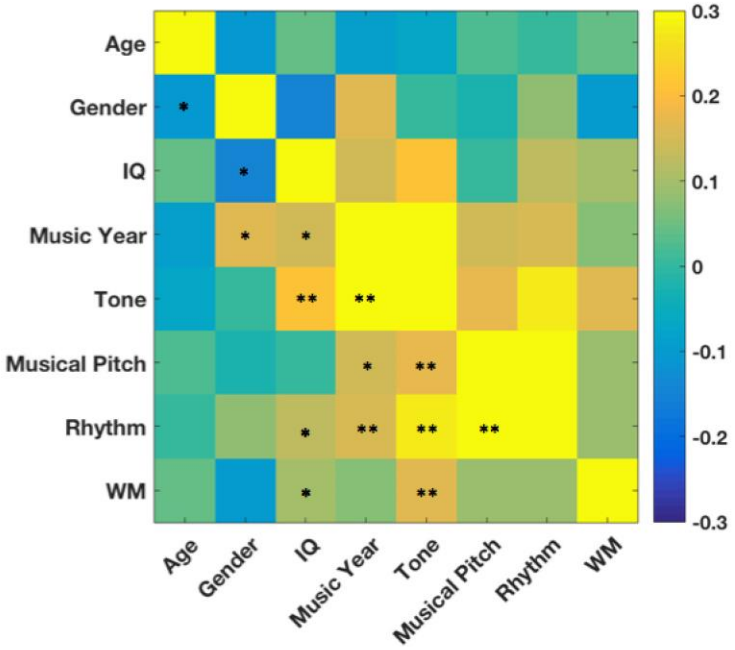

Fig. S2. Spearman's correlations of demographic variables and performance on the four behavioural tasks. *p < .05 (uncorrected) **p < .05 (corrected)

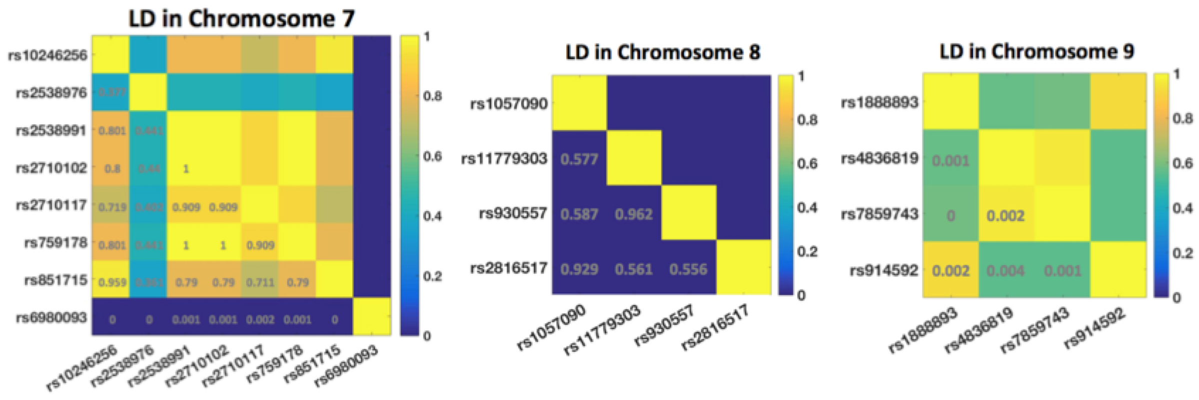

Fig. S3. Linkage disequilibrium (LD) among SNPs on the same chromosome. r^2 values were color-coded.

REFERENCES AND NOTES

1. D. M. Eberhard, G. F. Simons, C. D. Fennig, *Ethnologue: Languages of the World* (SIL International, 2019), vol. 22; http://www.ethnologue.com.

2. M. Haspelmath, M. Dryer, D. Gil, B. Comrie, *The World Atlas of Language Structures* (Oxford Univ. Press, Oxford, 2005).

3. D. Dediu, D. R. Ladd, Linguistic tone is related to the population frequency of the adaptive haplogroups of two brain size genes, *ASPM* and *Microcephalin*. *Proc. Natl. Acad. Sci. U. S. A.* **104**, 10944–10949 (2007).

4. D. Dediu, Are languages really independent from genes? If not, what would a genetic bias affecting language diversity look like? *Hum. Biol.* **83**, 279–296 (2011).

5. A. Baronchelli, N. Chater, R. Pastor-Satorras, M. H. Christiansen, The biological origin of linguistic diversity. *PLOS ONE* **7**, e48029 (2012).

6. M. Dryer, M. Haspelmath, *The World Atlas of Language Structures Online* (Max Planck Digital Library, 2011).

7. K. Brunk, B. Vernay, E. Griffith, N. L. Reynolds, D. Strutt, P. W. Ingham, A. P. Jackson, Microcephalin coordinates mitosis in the syncytial Drosophila embryo. *J. Cell Sci.* **120**, 3578–3588 (2007).

8. X. Zhong, L. Liu, A. Zhao, G. P. Pfeifer, X. Xu, The abnormal spindle-like, microcephaly-associated (ASPM) gene encodes a centrosomal protein. *Cell Cycle* **4**, 1227–1229 (2005).

9. J. Bond, E. Roberts, G. H. Mochida, D. J. Hampshire, S. Scott, J. M. Askham, K. Springell, M. Mahadevan, Y. J. Crow, A. F. Markham, C. A. Walsh, C. G. Woods, ASPM is a major determinant of cerebral cortical size. *Nat. Genet.* **32**, 316–320 (2002).

10. J. L. Fish, Y. Kosodo, W. Enard, S. Pääbo, W. B. Huttner, Aspm specifically maintains symmetric proliferative divisions of neuroepithelial cells. *Proc. Natl. Acad. Sci. U.S.A.* **103**, 10438–10443 (2006).

11. J. Cox, A. P. Jackson, J. Bond, C. G. Woods, What primary microcephaly can tell us about brain growth. *Trends Mol. Med.* **12**, 358–366 (2006).

12. B. L. Tang, Molecular genetic determinants of human brain size. *Biochem. Biophys. Res. Commun.* **345**, 911–916 (2006).

13. A. P. Jackson, D. P. McHale, D. A. Campbell, H. Jafri, Y. Rashid, J. Mannan, G. Karbani, P. Corry, M. I. Levene, R. F. Mueller, A. F. Markham, N. J. Lench, C. G. Woods, Primary autosomal recessive microcephaly (MCPH1) maps to chromosome 8p22-pter. *Am. J. Hum. Genet.* **63**, 541–546 (1998).

14. A. P. Jackson, H. Eastwood, S. M. Bell, J. Adu, C. Toomes, I. M. Carr, E. Roberts, D. J. Hampshire, Y. J. Crow, A. J. Mighell, G. Karbani, H. Jafri, Y. Rashid, R. F. Mueller, A.

F. Markham, C. G. Woods, Identification of microcephalin, a protein implicated in determining the size of the human brain. *Am. J. Hum. Genet.* **71**, 136–142 (2002).

15. N. Mekel-Bobrov, S. L. Gilbert, P. D. Evans, E. J. Vallender, J. R. Anderson, R. R. Hudson, S. A. Tishkoff, B. T. Lahn, Ongoing adaptive evolution of ASPM, a brain size determinant in Homo sapiens. *Science* **309**, 1720–1722 (2005).

16. P. D. Evans, S. L. Gilbert, N. Mekel-Bobrov, E. J. Vallender, J. R. Anderson, L. M. Vaez-Azizi, S. A. Tishkoff, R. R. Hudson, B. T. Lahn, Microcephalin, a gene regulating brain size, continues to evolve adaptively in humans. *Science* **309**, 1717–1720 (2005).

17. N. Mekel-Bobrov, D. Posthuma, S. L. Gilbert, P. Lind, M. F. Gosso, M. Luciano, S. E. Harris, T. C. Bates, T. J. C. Polderman, L. J. Whalley, H. Fox, J. M. Starr, P. D. Evans, G. W. Montgomery, C. Fernandes, P. Heutink, N. G. Martin, D. I. Boomsma, I. J. Deary, M. J. Wright, E. J. C. de Geus, B. T. Lahn, The ongoing adaptive evolution of *ASPM* and *Microcephalin* is not explained by increased intelligence. *Hum. Mol. Genet.* **16**, 600–608 (2007).

18. C. Dobson-Stone, J. M. Gatt, S. A. Kuan, S. M. Grieve, E. Gordon, L. M. Williams, P. R. Schofield, Investigation of *MCPH1* G37995C and *ASPM* A44871G polymorphisms and brain size in a healthy cohort. *Neuroimage* **37**, 394–400 (2007).

19. N. Timpson, J. Heron, G. D. Smith, W. Enard, Comment on papers by Evans et al. and Mekel-Bobrov et al. on evidence for positive selection of MCPH1 and ASPM. *Science* **317**, 1036 (2007).

20. S. Passemard, A. Verloes, T. Billette de Villemeur, O. Boespflug-Tanguy, K. Hernandez, M. Laurent, B. Isidor, C. Alberti, N. Pouvreau, S. Drunat, B. Gérard, V. El Ghouzzi, J. Gallego, M. Elmaleh-Bergès, W. B. Huttner, S. Eliez, P. Gressens, M. Schaer, Abnormal spindle-like microcephaly-associated (*ASPM*) mutations strongly disrupt neocortical structure but spare the hippocampus and long-term memory. *Cortex* **74**, 158–176 (2016).

21. D. Bendor, X. Wang, The neuronal representation of pitch in primate auditory cortex. *Nature* **436**, 1161–1165 (2005).

22. P. C. M. Wong, C. M. Warrier, V. B. Penhune, A. K. Roy, A. Sadehh, T. B. Parrish, R. J. Zatorre, Volume of left Heschl's Gyrus and linguistic pitch learning. *Cereb. Cortex* **18**, 828–836 (2008).

23. P. C. M. Wong, B. Chandrasekaran, J. Zheng, The derived allele of *ASPM* is associated with lexical tone perception. *PLOS ONE* **7**, e34243 (2012).

24. I. Maddieson, Tone, in *The World Atlas of Language Structures Online*, M. S. Dryer, M. S. and M. Haspelmath, Eds. (Max Planck Institute for Evolutionary Anthropology, 2013).

25. L. M. Rimol, I. Agartz, S. Djurovic, A. A. Brown, J. C. Roddey, A. K. Kähler, M. Mattingsdal, L. Athanasiu, A. H. Joyner, N. J. Schork, E. Halgren, K. Sundet, I. Melle,

A. M. Dale, O. A. Andreassen; Alzheimer's Disease Neuroimaging Initiative, Sex-dependent association of common variants of microcephaly genes with brain structure. *Proc. Natl. Acad. Sci. U. S. A.* **107**, 384–388 (2010).

26. J.-K. Wang, Y. Li, B. Su, A common SNP of MCPH1 is associated with cranial volume variation in Chinese population. *Hum. Mol. Genet.* **17**, 1329–1335 (2008).

27. P. C. M. Wong, V. Ciocca, A. H. D. Chan, L. Y. Y. Ha, L.-H. Tan, I. Peretz, Effects of culture on musical pitch perception. *PLOS ONE* **7**, e33424 (2012).

28. S. C. Vernes, D. F. Newbury, B. S. Abrahams, L. Winchester, J. Nicod, M. Groszer, M. Alarcón, P. L. Oliver, K. E. Davies, D. H. Geschwind, A. P. Monaco, S. E. Fisher, A functional genetic link between distinct developmental language disorders. *N. Engl. J. Med.* **359**, 2337–2345 (2008).

29. D. F. Newbury, L. Winchester, L. Addis, S. Paracchini, L.-L. Buckingham, A. Clark, W. Cohen, H. Cowie, K. Dworzynski, A. Everitt, I. M. Goodyer, E. Hennessy, A. D. Kindley, L. L. Miller, J. Nasir, A. O'Hare, D. Shaw, Z. Simkin, E. Simonoff, V. Slonims, J. Watson, J. Ragoussis, S. E. Fisher, J. R. Seckl, P. J. Helms, P. F. Bolton, A. Pickles, G. Conti-Ramsden, G. Baird, D. V. M. Bishop, A. P. Monaco, *CMIP* and *ATP2C2* modulate phonological short-term memory in language impairment. *Am. J. Hum. Genet.* **85**, 264–272 (2009).

30. P. C. M. Wong, M. Ettlinger, J. Zheng, Linguistic grammar learning and DRD2-TAQ-IA polymorphism. *PLOS ONE* **8**, e64983 (2013).

31. P. C. M. Wong, K. Morgan-Short, M. Ettlinger, J. Zheng, Linking neurogenetics and individual differences in language learning: The dopamine hypothesis. *Cortex* **48**, 1091–1102 (2012).

32. P. C. M. Wong, T. K. Perrachione, Learning pitch patterns in lexical identification by native English-speaking adults. *Appl. Psycholinguist.* **28**, 565–585 (2007).

33. C. L. Caldwell-Harris, A. Lancaster, D. Robert Ladd, D. Dediu, M. H. Christiansen, Factors influencing sensitivity to lexical tone in an artificial language: Implications for second language learning. *Stud. Second Lang. Acquis*. **37**, 335–357 (2015).

34. F. Vargha-Khadem, K. Watkins, K. Alcock, P. Fletcher, R. Passingham, Praxic and nonverbal cognitive deficits in a large family with a genetically transmitted speech and language disorder. *Proc. Natl. Acad. Sci. U.S.A.* **92**, 930–933 (1995).

35. C. M. Stein, B. Truitt, F. Deng, A. A. Ciesla, F. Qiu, P. Joseph, R. Raghavendra, J. Fondran, R. P. Igo Jr, J. Tag, L. Freebairn, H. G. Taylor, B. A. Lewis, S. K. Iyengar, Association between AVPR1A, DRD2, and ASPM and endophenotypes of communication disorders. *Psychiatr. Genet.* **24**, 191–200 (2014).

36. dbSNP: Database for Short Genetic Variations, National Center for Biotechnology Information, National Library of Medicine, National Institutes of Health, US Department of Health and Human Services; https://www.ncbi.nlm.nih.gov/snp/.

37. P. C. M. Wong, E. Skoe, N. M. Russo, T. Dees, N. Kraus, Musical experience shapes human brainstem encoding of linguistic pitch patterns. *Nat. Neurosci.* **10**, 420–422 (2007).

38. A. Okbay, J. P. Beauchamp, M. A. Fontana, J. J. Lee, T. H. Pers, C. A. Rietveld, P. Turley, G.-B. Chen, V. Emilsson, S. F. W. Meddens, S. Oskarsson, J. K. Pickrell, K. Thom, P. Timshel, R. de Vlaming, A. Abdellaoui, T. S. Ahluwalia, J. Bacelis, C. Baumbach, G. Bjornsdottir, J. H. Brandsma, M. P. Concas, J. Derringer, N. A. Furlotte, T. E. Galesloot, G. Girotto, R. Gupta, L. M. Hall, S. E. Harris, E. Hofer, M. Horikoshi, J. E. Huffman, K. Kaasik, I. P. Kalafati, R. Karlsson, A. Kong, J. Lahti, S. J. van der Lee, C. deLeeuw, P. A. Lind, K.-O. Lindgren, T. Liu, M. Mangino, J. Marten, E. Mihailov, M. B. Miller, P. J. van der Most, C. Oldmeadow, A. Payton, N. Pervjakova, W. J. Peyrot, Y. Qian, O. Raitakari, R. Rueedi, E. Salvi, B. Schmidt, K. E. Schraut, J. Shi, A. V. Smith, R. A. Poot, B. St Pourcain, A. Teumer, G. Thorleifsson, N. Verweij, D. Vuckovic, J. Wellmann, H.-J. Westra, J. Yang, W. Zhao, Z. Zhu, B. Z. Alizadeh, N. Amin, A. Bakshi, S. E. Baumeister, G. Biino, K. Bønnelykke, P. A. Boyle, H. Campbell, F. P. Cappuccio, G. Davies, J.-E. De Neve, P. Deloukas, I. Demuth, J. Ding, P. Eibich, L. Eisele, N. Eklund, D. M. Evans, J. D. Faul, M. F. Feitosa, A. J. Forstner, I. Gandin, B. Gunnarsson, B. V. Halldórsson, T. B. Harris, A. C. Heath, L. J. Hocking, E. G. Holliday, G. Homuth, M. A. Horan, J.-J. Hottenga, P. L. de Jager, P. K. Joshi, A. Jugessur, M. A. Kaakinen, M. Kähönen, S. Kanoni, L. Keltigangas-Järvinen, L. A. L. M. Kiemeney, I. Kolcic, S. Koskinen, A. T. Kraja, M. Kroh, Z. Kutalik, A. Latvala, L. J. Launer, M. P. Lebreton, D. F. Levinson, P. Lichtenstein, P. Lichtner, D. C. M. Liewald; LifeLines Cohort Study, A. Loukola, P. A. Madden, R. Mägi, T. Mäki-Opas, R. E. Marioni, P. Marques-Vidal, G. A. Meddens, G. McMahon, C. Meisinger, T. Meitinger, Y. Milaneschi, L. Milani, G. W. Montgomery, R. Myhre, C. P. Nelson, D. R. Nyholt, W. E. R. Ollier, A. Palotie, L. Paternoster, N. L. Pedersen, K. E. Petrovic, D. J. Porteous, K. Räikkönen, S. M. Ring, A. Robino, O. Rostapshova, I. Rudan, A. Rustichini, V. Salomaa, A. R. Sanders, A.-P. Sarin, H. Schmidt, R. J. Scott, B. H. Smith, J. A. Smith J. A. Staessen, E. Steinhagen-Thiessen, K. Strauch, A. Terracciano, M. D. Tobin, S. Ulivi, S. Vaccargiu, L. Quaye, F. J. A. van Rooij, C. Venturini, A. A. E. Vinkhuyzen, U. Völker, H. Völzke, J. M. Vonk, D. Vozzi, J. Waage, E. B. Ware, G. Willemsen, J. R. Attia, D. A. Bennett, K. Berger, L. Bertram, H. Bisgaard, D. I. Boomsma, I. B. Borecki, U. Bültmann, C. F. Chabris, F. Cucca, D. Cusi, I. J. Deary, G. V. Dedoussis, C. M. van Duijn, J. G. Eriksson, B. Franke, L. Franke, P. Gasparini, P. V. Gejman, C. Gieger, H.-J. Grabe, J. Gratten, P. J. F. Groenen, V. Gudnason, P. van der Harst, C. Hayward, D. A. Hinds, W. Hoffmann, E. Hyppönen, W. G. Iacono, B. Jacobsson, M.-R. Järvelin, K.-H. Jöckel, J. Kaprio, S. L. R. Kardia, T. Lehtimäki, S. F. Lehrer, P. K. E. Magnusson, N. G. Martin, M. McGue, A. Metspalu, N. Pendleton, B. W. J. H. Penninx, M. Perola, N. Pirastu, M. Pirastu, O. Polasek, D. Posthuma, C. Power, M. A. Province, N. J. Samani, D. Schlessinger, R. Schmidt, T. I. A. Sørensen, T. D. Spector, K. Stefansson, U. Thorsteinsdottir, A. R.

Thurik, N. J. Timpson, H. Tiemeier, J. Y. Tung, A. G. Uitterlinden, V. Vitart, P. Vollenweider, D. R. Weir, J. F. Wilson, A. F. Wright, D. C. Conley, R. F. Krueger, G. Davey Smith, A. Hofman, D. I. Laibson, S. E. Medland, M. N. Meyer, J. Yang, M. Johannesson, P. M. Visscher, T. Esko, P. D. Koellinger, D. Cesarini, D. J. Benjamin, Genome-wide association study identifies 74 loci associated with educational attainment. *Nature* **533**, 539–542 (2016).

39. N. C. Battle, S. Choudhry, H.-J. Tsai, C. Eng, G. Kumar, K. B. Beckman, M. Naqvi, K. Meade, H. G. Watson, M. Lenoir, E. G. Burchard; SAGE Investigators, Ethnicity-specific gene–gene interaction between IL-13 and IL-4Rα among African Americans with asthma. *Am. J. Respir. Crit. Care Med.* **175**, 881–887 (2007).

40. H. S. Kim, D. K. Sherman, J. Y. Sasaki, J. Xu, T. Q. Chu, C. Ryu, E. M. Suh, K. Graham, S. E. Taylor, Culture, distress, and oxytocin receptor polymorphism (OXTR) interact to influence emotional support seeking. *Proc. Natl. Acad. Sci. U.S.A.* **107**, 15717–15721 (2010).

41. J. Y. Chiao, K. D. Blizinsky, Culture–gene coevolution of individualism–collectivism and the serotonin transporter gene. *Proc. Biol. Sci.* **277**, 529–537 (2010).

42. L. Lee, H. C. Nusbaum, Processing interactions between segmental and suprasegmental information in native speakers of English and Mandarin Chinese. *Percept. Psychophys.* **53**, 157–165 (1993).

43. B. H. Repp, H.-B. Lin, Integration of segmental and tonal information in speech perception: A cross-linguistic study. *J. Phon.* **18**, 481–495 (1990).

44. E. Dupoux, C. Pallier, N. Sebastian, J. Mehler, A destressing "deafness" in French? *J. Mem. Lang.* **36**, 406–421 (1997).

45. E. Dupoux, N. Sebastián-Gallés, E. Navarrete, S. Peperkamp, Persistent stress 'deafness': The case of French learners of Spanish. *Cognition* **106**, 682–706 (2008).

46. H. Rahmani, T. Rietveld, C. Gussenhoven, Stress "deafness" reveals absence of lexical marking of stress or tone in the adult grammar. *PLOS ONE* **10**, e0143968 (2015).

47. A. M.-Y. Wong, V. Ciocca, S. Yung, The perception of lexical tone contrasts in Cantonese children with and without specific language impairment (SLI). *J. Speech Lang. Hear. Res.* **52**, 1493–1509 (2009).

48. X. Wang, S. Wang, Y. Fan, D. Huang, Y. Zhang, Speech-specific categorical perception deficit in autism: An Event-related potential study of lexical tone processing in Mandarin-speaking children. *Sci. Rep.* **7**, 43254 (2017).

49. S. Seshadri, A. L. Fitzpatrick, M. A. Ikram, A. L. DeStefano, V. Gudnason, M. Boada, J. C. Bis, A. V. Smith, M. M. Carassquillo, J. C. Lambert, D. Harold, E. M. C. Schrijvers, R. Ramirez-Lorca, S. Debette, W. T. Longstreth Jr, A. C. J. W. Janssens, V. S. Pankratz, J. F. Dartigues, P. Hollingworth, T. Aspelund, I. Hernandez, A. Beiser, L. H. Kuller, P. J.

Koudstaal, D. W. Dickson, C. Tzourio, R. Abraham, C. Antunez, Y. Du, J. I. Rotter, Y. S. Aulchenko, T. B. Harris, R. C. Petersen, C. Berr, M. J. Owen, J. Lopez-Arrieta, B. N. Varadarajan, J. T. Becker, F. Rivadeneira, M. A. Nalls, N. R. Graff-Radford, D. Campion, S. Auerbach, K. Rice, A. Hofman, P. V. Jonsson, H. Schmidt, M. Lathrop, T. H. Mosley, R. Au, B. M. Psaty, A. G. Uitterlinden, L. A. Farrer, T. Lumley, A. Ruiz, J. Williams, P. Amouyel, S. G. Younkin, P. A. Wolf, L. J. Launer, O. L. Lopez, C. M. van Duijn, M. M. B. Breteler; CHARGE Consortium; GERAD1 Consortium; EADI1 Consortium, Genome-wide analysis of genetic loci associated with Alzheimer disease. *JAMA* **303**, 1832–1840 (2010).

50. J. Wang, Y. Tao, F. Song, Y. Sun, J. Ott, D. Saffen, Common regulatory variants of CYFIP1 contribute to susceptibility for autism spectrum disorder (ASD) and classical autism. *Ann. Hum. Genet.* **79**, 329–340 (2015).

51. I. I. Gottesman, J. Shields, Genetic theorizing and schizophrenia. *Br. J. Psychiatry* **122**, 15–30 (1973).

52. C. McDonald, E. T. Bullmore, P. C. Sham, X. Chitnis, H. Wickham, E. Bramon, R. M. Murray, Association of genetic risks for schizophrenia and bipolar disorder with specific and generic brain structural endophenotypes. *Arch. Gen. Psychiatry* **61**, 974–984 (2004).

53. M. Losh, G. E. Martin, M. Lee, J. Klusek, J. Sideris, S. Barron, T. Wassink, Developmental markers of genetic liability to autism in parents: A longitudinal, multigenerational study. *J. Autism Dev. Disord.* **47**, 834–845 (2017).

54. J. B. Misyak, M. H.Christiansen, Genetic variation and individual differences in language, in *Experience, Variation, and Generalization: Learning A First Language*, I. Arnon, E. V. Clark, Eds. (Trends in Language Acquisition Research, Amsterdam: John Benjamins, 2011), vol. 7, pp.223–238.

55. P. C. M. Wong, L. C. Vuong, K. Liu, Personalized learning: From neurogenetics of behaviors to designing optimal language training. *Neuropsychologia* **98**, 192–200 (2017).

56. C. Everett, D. E. Blasi, S. G. Roberts, Climate, vocal folds, and tonal languages: Connecting the physiological and geographic dots. *Proc. Natl. Acad. Sci. U.S.A.* **112**, 1322–1327 (2015).

57. L. Brown, R. Sherbenou, S. Johnsen, *TONI 4, Test of Nonverbal Intelligence* (Pro-Ed, Austin, 2010).

58. J. M. Broadway, R. W. Engle, Validating running memory span: Measurement of working memory capacity and links with fluid intelligence. *Behav. Res. Methods* **42**, 563–570 (2010).

59. S. Gabriel, L. Ziaugra, D. Tabbaa, SNP genotyping using the Sequenom MassARRAY iPLEX platform. *Curr. Protoc. Hum. Genet.* **Chapter 2**, 2.12.1–1.12.18 (2009).

60. S. Purcell, B. Neale, K. Todd-Brown, L. Thomas, M. A. R. Ferreira, D. Bender, J. Maller, P. Sklar, P. I. W. de Bakker, M. J. Daly, P. C. Sham, PLINK: A tool set for whole-genome association and population-based linkage analyses. *Am. J. Hum. Genet.* **81**, 559–575 (2007).

61. F. Pedregosa, G. Varoquaux, A. Gramfort, V. Michel, B. Thirion, O. Grisel, M. Blondel, P. Prettenhofer, R. Weiss, V. Dubourg, J. Vanderplas, A. Passos, D. Cournapeau, M. Brucher, M. Perrot, É. Duchesnay, Scikit-learn: Machine Learning in Python. *J. Mach. Learn. Res.* **12**, 2825–2830 (2011).

62. Y.-J. Zhao, Y.-P. Wang, W.-Z. Yang, H.-W. Sun, H.-W. Ma, Y.-R. Zhao, *CNTNAP2* is significantly associated with speech sound disorder in the chinese han population. *J. Child Neurol.* **30**, 1806–1811 (2015).

63. A. J. O. Whitehouse, D. V. M. Bishop, Q. W. Ang, C. E. Pennell, S. E. Fisher, *CNTNAP2* variants affect early language development in the general population. *Genes Brain Behav.* **10**, 451–456 (2011).

64. N. Li, C. W. Bartlett, Defining the genetic architecture of human developmental language impairment. *Life Sci.* **90**, 469–475 (2012).

65. N. Zink, W. Bensmann, L. Arning, L. S. Colzato, A.-K. Stock, C. Beste, The role of DRD1 and DRD2 receptors for response selection under varying complexity levels: Implications for metacontrol processes. *Int. J. Neuropsychopharmacol.* **22**, 747–753 (2019).

66. S. H. Kollins, A. D. Anastopoulos, A. M. Lachiewicz, D. FitzGerald, E. Morrissey-Kane, M. E. Garrett, S. L. Keatts, A. E. Ashley-Koch, SNPs in dopamine D2 receptor gene (DRD2) and norepinephrine transporter gene (NET) are associated with continuous performance task (CPT) phenotypes in ADHD children and their families. *Am. J. Med. Genet. B Neuropsychiatr. Genet.* **147B**, 1580–1588 (2008).

67. Y. Zhang, A. Bertolino, L. Fazio, G. Blasi, A. Rampino, R. Romano, M.-L. T. Lee, T. Xiao, A. Papp, D. Wang, W. Sadée, Polymorphisms in human dopamine D2 receptor gene affect gene expression, splicing, and neuronal activity during working memory. *Proc. Natl. Acad. Sci. U.S.A.* **104**, 20552–20557 (2007).

68. M. Aguilera, N. Barrantes-Vidal, B. Arias, J. Moya, H. Villa, M. I. Ibáñez, M. A. Ruipérez, G. Ortet, L. Fañanás, Putative role of the COMT gene polymorphism (Val158Met) on verbal working memory functioning in a healthy population. *Am. J. Med. Genet. B Neuropsychiatr. Genet.* **147B**, 898–902 (2008).

69. Y. Wang, J. Li, C. Chen, C. Chen, B. Zhu, R. K. Moysis, X. Lei, H. Li, Q. Liu, D. Xiu, B. Liu, W. Chen, G. Xue, Q. Dong, COMT rs4680 Met is not always the 'smart allele': Val allele is associated with better working memory and larger hippocampal volume in healthy Chinese. *Genes Brain Behav.* **12**, 323–329 (2013).

70. C. Wu, Y. Ding, B. Chen, Y. Gao, Q. Wang, Z. Wu, L. Lu, L. Luo, C. Zhang, X. Bao, P. Yang, L. Fan, M. Lei, L. Li, Both Val158Met polymorphism of catechol-O-methyltransferase gene and menstrual cycle affect prepulse inhibition but not attentional modulation of prepulse inhibition in younger-adult females. *Neuroscience* **404**, 396–406 (2019).

71. S. Zhang, J. Zhao, Z. Guo, J. A. Jones, P. Liu, H. Liu, The association between genetic variation in FOXP2 and sensorimotor control of speech production. *Front. Neurosci.* **12**, 666 (2018).
